# Exploring engagement with a web-based dietary intervention for adults with type 2 diabetes: A mixed methods evaluation of the T2Diet study

**DOI:** 10.1371/journal.pone.0279466

**Published:** 2022-12-30

**Authors:** Jedha Dening, Karly Zacharia, Kylie Ball, Elena S. George, Sheikh Mohammed Shariful Islam

**Affiliations:** 1 Institute for Physical Activity and Nutrition, School of Exercise and Nutrition Sciences, Deakin University, Melbourne Burwood, Victoria, Australia; 2 Faculty of Health & Medicine, School of Health Sciences, University of Newcastle, Callaghan, New South Wales, Australia; University of Gondar, ETHIOPIA

## Abstract

**Background:**

Improved understanding of participant engagement in web-based dietary interventions is needed. Engagement is a complex construct that may be best explored through mixed methods to gain comprehensive insight. To our knowledge, no web-based dietary intervention in people with type 2 diabetes (T2D) has previously used a mixed methods approach. The aim of this study was to explore factors that may contribute to effective engagement in a web-based dietary program for people with T2D.

**Methods:**

This study employed a mixed methods intervention design, with a convergent design embedded for post-intervention evaluation. The convergent design collected and analyzed quantitative and qualitative data independent of each other, with the two datasets merged/compared during results/interpretation. Quantitative data collected from intervention group participants (n = 40) were self-administered questionnaires and usage data with average values summarized. Qualitative data were participant semi-structured interviews (n = 15) incorporating a deductive-inductive thematic analysis approach.

**Results:**

The results from the quantitative and qualitative data indicated positive overall engagement with the web-based dietary program. Factors that contributed to effective engagement were sustained frequency and intensity of engagement; structured weekly program delivery; participants affective engagement prior to and during the intervention, with positive affective states enhancing cognitive and behavioral engagement; and participants experience of value and reward. In addition, the user-centered development process employed prior to intervention delivery played an important role in facilitating positive engagement outcomes.

**Conclusion:**

This study yielded novel findings by integrating qualitative and quantitative data to explore engagement with a web-based dietary program involving people with T2D. Effective engagement occurred in this intervention through a combination of factors related to usage and participants’ affective, cognitive and behavioral states. The engagement outcomes that emerged will be useful to current and future researchers using digital technologies to deliver lifestyle interventions for T2D or other chronic health conditions.

## Introduction

Emerging evidence suggests that web-based interventions can be effective for providing dietary self-management support to people with T2D [1]. Intervention effectiveness is thought to be improved via participant engagement [2]. Evidence among digital glucose monitoring apps suggest engagement may lead to improved health outcomes in people with T2D [3]. Currently however, little is understood about engagement in web-based behavioral or dietary interventions [4, 5]. This is largely due to confusion with other terms such as adherence, along with lack of measurement and reporting of engagement [2, 4–9]. It is widely recognized that engagement is a complex construct involving cognitive, affective and behavioral components [6, 10], and encompassing both micro- and- macro levels [2, 7]. Cognitive engagement relates to the process of acquiring knowledge, making decisions and producing responses; affective engagement relates to feelings, attitudes and moods; and behavioral engagement relates to reactions or actions made in response to program stimuli [2, 6, 7]. Micro level engagement refers to website usage, user experience and aspects of moment-to-moment engagement [2]. Macro level engagement refers to involvement and motivation with the behavior change process [2]. With the field of engagement in web-based behavioral or dietary interventions still in its infancy, further exploration of these constructs and an increased understanding of intervention aspects that may facilitate effective engagement are needed [2, 5, 7, 11].

In terms of the constructs of engagement, reviews identified that published evaluations of eHealth interventions have largely focused on behavioral engagement, rather than exploring affective and cognitive components [10]. Authors have suggested that usage data alone cannot capture the relationship between complex engagement constructs [6, 12]. Instead, the addition of qualitative methods to capture insights about user experience and offline engagement in the behavior change process may be important [2, 7]. It has been noted however, that taking a mixed methods approach to explore engagement has rarely been conducted [2, 10]. Using mixed methods can be a complex process [13]. However, the benefit is achieving breadth and depth in understanding [2, 13]. To the best of our knowledge, no web-based dietary intervention in people with T2D has previously used a mixed methods approach to investigate engagement. The aim of this study was to explore factors that may contribute to effective engagement in a web-based dietary program for people with T2D.

## Material and methods

### Study design

**Fig 1:**
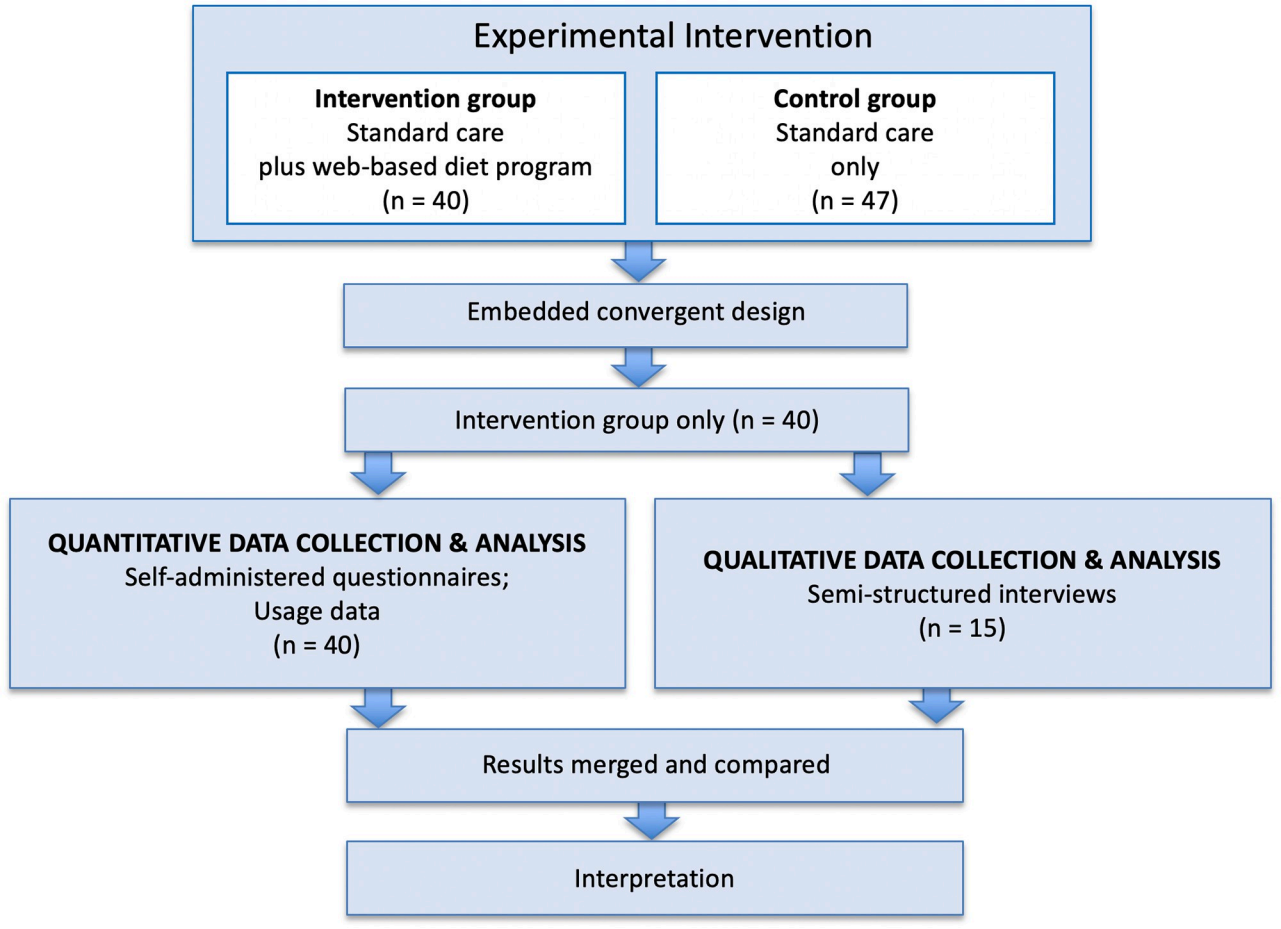
Overview of the mixed methods intervention design, with embedded convergent design.

This study employed a mixed methods intervention design, with a convergent design embedded for post-intervention evaluation (Fig 1) [13]. The convergent design applied in this study incorporated quantitative and qualitative data, which were collected and analyzed independent of each other. The centerpiece of a convergent design is merging/comparing the two datasets during results/interpretation [13, 14]. Thus, the ‘mixing’ of the datasets occurred by comparing/combining the results and by merging during interpretation/discussion [13]. Pragmatism was the methodological umbrella applied, which posits that answering the research question via ‘what works’ is of primary importance [13, 14].

### T2Diet study background

**Fig 2:**
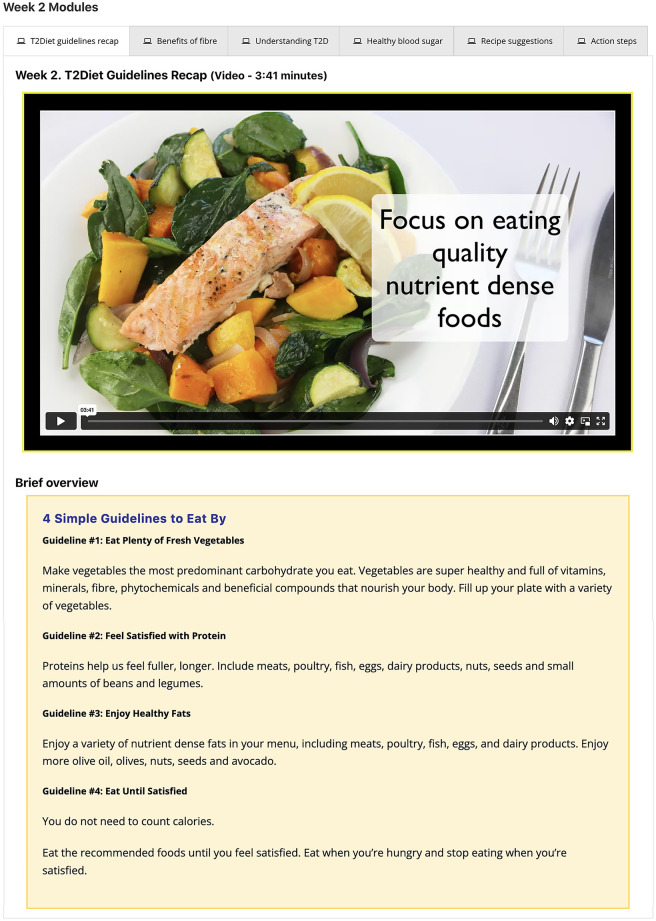
Example presentation of the weekly structured modules.

Effective engagement is said to begin with an iterative user-centered process employed during intervention development stages [6, 7]. Thus, four phases of iterative user-centered development, involving adults with T2D, were conducted for the T2Diet study, details were published elsewhere [15]. Furthermore, the study protocol detailing the intervention was published elsewhere [16]. In brief, the intervention was a 16-week theoretically-informed automated web-based dietary program for adults with T2D. Intervention participants received weekly structured behavior change modules consisting of videos, informational summaries, resource links, recipes and action steps (Fig 2), along with on-demand resources. The structured modules were automated to be delivered to participants sequentially, on a weekly basis over the 16 weeks. In addition, participants received twice-weekly email notifications.

### Participants

Trial participants were adults aged 40 to 89 years with self-reported T2D diagnosis and hemoglobin A1c levels ≥7.0% within six months of enrolment, located in Australia with internet and email access. All eligible participants meeting the inclusion criteria [16] were enrolled in the study, regardless of the duration of their diabetes. Quantitative data was collected from all intervention group participants who completed the study (n = 40). Qualitative data was collected from a convenience sample [17] recruited from the pool of intervention group participants who completed the study (n = 15). A qualitative sample size of 8–20 was decided *a priori* as adequate to achieve data saturation [18]. Written informed consent was obtained from all participants for entry into the trial, and to be contacted for participation in the post-intervention interviews. Ethics approval was obtained from Deakin University Human Research Ethics Committee (#2020–349).

### Quantitative methods

Two self-administered online questionnaires were completed by participants post-intervention (S1 Appendix): 1) the User Engagement Scale-short form (UES-SF) [19], and 2) the Honeycomb Model [20, 21]. The UES-SF has been validated for use in assessing digital technologies across Western adult populations [19]. The UES-SF measures engagement across four domains: aesthetic appeal, focused attention, perceived usability and reward. Each of the four domains have three questions, which were collected using a 5-point rating scale from 1 (strongly disagree) to 5 (strongly agree). Average scores for the four domains were calculated by dividing the total score of the three questions in each domain by three. The Honeycomb Model measures user experience across seven domains: usefulness, credible, desirable, usable, findable, accessible and valuable. Each of the seven domains has one question, which were collected on an 8-point rating scale from 0 (worst experience) to 8 (best experience). Average scores for each of the seven domains were calculated. The Honeycomb Model is not a validated scale but was originally designed as an online business tool [20]. In research, it is a new instrument that has typically been used in the process of designing behavioral interventions or in usability testing [22, 23]. Thus, taking the model into post-intervention evaluation of engagement is a novel approach. The UES-SF and the Honeycomb Model do not provide guidance for analyzing results. Rather, these new instruments are designed for comparison within studies (for example, doing multiple assessments with the instruments at different time points); or comparison across studies (for example, as more studies implement these instruments, comparisons between scores and domains can be made).

Aggregate usage data for the intervention period included average session duration and type of device. Email open and click through rates were collected from the two weekly email notifications and averages were calculated. Number of participant logins for each week of the intervention were collected and average logins per week were calculated. Dropout attrition was defined as losing participants to follow-up (non-completion of post-intervention questionnaires), while nonusage attrition was defined as participants who failed to use the intervention as intended [24], which was operationalized *a priori* that participants would login to the website at least once per week to access new modules.

### Qualitative methods

Participants who had provided consent at enrolment to attend a post-intervention interview, were invited via email. Participants who accepted the invitation were engaged in 20 to 30-minute semi-structured individual interviews. The semi-structured interview guide (S2 Appendix) had previously been tested during our user-centered development work [15], and was revised for this study with pilot testing through discussion (JD, SMSI) prior to the interviews. Phone interviews were conducted by the same facilitator (JD), and audio recorded with participant’s written and verbal consent. Field notes were logged after each interview to support an assessment of adequate data saturation. Under the methodological umbrella of pragmatism [13, 14] and a process of interpretive judgement [18], saturation was assessed to be reached after interviewing 15 participants, where information power—defined in this study as information quality in relation to the research question [25]; and repetition of responses was indicated in the field notes [18]. Audio recordings were transcribed verbatim and anonymized for confidentiality.

### Analysis

The study sample was described using descriptive statistics (mean and percentage). As described above, self-administered questionnaires and usage data were summarized using average values. Average scores for the Honeycomb Model were visually summarized with the User Experience Radar [26]. For qualitative analysis, a deductive categorization framework [27] provided a template of predefined categories (S3 Appendix) consisting of the three constructs of engagement (affective, cognitive and behavioral) [2], and two additional categories (behavior changes and intervention features) to bolster understanding of effective engagement [10]. Braun and Clarke’s [28] six-step inductive thematic analysis was used to generate codes/themes under each of the predefined categories. Analysis was conducted independently (KZ) using NVivo (Version 12). Codes and themes that emerged were then discussed and revised by two researchers (JD, KZ), followed by feedback from the research team.

### Research team

In qualitative research, researchers are instruments in the data collection and analysis process [29]. Our multidisciplinary research team provided a broad set of skills, beliefs and perspectives to the process of planning, implementing and analyzing the research [30–34].

### Data management and integrity

Due to competing interests (JD), monitoring and robust systems were implemented across the course of the study. Monthly monitoring with a team of three supervisors and the associate head of school occurred. Master files were downloaded by the principal investigator (SMSI), deposited in safe storage and never modified. Copies were utilized by research team members as necessary. All data were cross-checked by the principal investigator or a research assistant. Qualitative analysis was conducted by an independent researcher, with the research team involved in interpretation of data.

## Results

For this mixed methods exploration of engagement, a total of 40 intervention participants provided quantitative data, and 15 intervention participants provided qualitative data. Participant characteristics are presented in Table 1. There were no divergences and inconsistencies between the two datasets. Results are presented below.

**Table 1:** Participant characteristics.

Demographic category	Demographic detail	Intervention group (n = 40)	Qualitative subgroup (n = 15)
Gender, n (%)	Female	23 (57)	8 (53)
	Male	17 (43)	7 (47)
Age (years), mean (SD)		61.3 (9.4)	63.9 (7.6)
Duration of T2D, n (%)	<1 year	8 (20)	2 (13)
	1–6 years	14 (36)	6 (40)
	7–15 years	14 (36)	5 (33)
	>15 years	4 (10)	2 (13)
Family history of T2D, n (%)	Yes	19 (47.5)	7 (47)
	No	21 (52.5)	8 (53)
Country of birth, n (%)	Australia	31 (78)	11 (73)
	International	8 (22)	4 (27)
Relationship status, n (%)	Married/living with a partner	31 (78)	11 (73)
	Separated, divorced, widowed	7 (18)	2 (20)
	Never married	2 (5)	1 (7)
Education level, n (%)	Bachelor’s degree or above	13 (33)	4 (27)
	Tertiary/trade certificate	13 (33)	3 (20)
	Completed high school	9 (23)	5 (33)
	None	5 (13)	3 (20)
Employment status, n (%)	Employed full-time	10 (25)	2 (13)
	Employed part-time	7 (18)	2 (13)
	Retired	17 (43)	7 (47)
	Unemployed	6 (17)	4 (27)

### Frequency of engagement

Average weekly login rates are presented in Table 2. Usage was highest in the first two weeks, dropped in the third week yet remained stable from weeks three to nine. From week 10 usage declined further, though remained at an average of one or more logins per week, except for week 15. Dropout attrition was 7.5% (n = 3), nonusage attrition was 12.5% (n = 5), with 80% of participants (n = 32) logging into the site at least once per week. For the intervention period, the site was accessed most frequently on desktop computer (451), followed by mobile phone (341) and tablet (51). The primary intervention feature consistently noted by participants as a facilitator of engagement was the structured weekly mode of delivery.

**Table 2:** Average logins per week.

Week	1	2	3	4	5	6	7	8	9	10	11	12	13	14	15	16
	6.2	3.2	1.9	1.6	1.7	1.6	1.7	1.5	1.5	1.3	1.0	1.2	1.1	1.3	0.2	1.1

“I looked forward to actually, you know, getting it each week, which was interesting,”

Participant 13.

### Intensity and duration of engagement

Email open rates (percentage of participants opening email notifications) were higher than email click through rates (percentage of participants clicking on links in email notifications), 80.8% and 42.2%, respectively. Email one elicited higher response than email two, 41.3% opens/25.8% clicks, 39.5% opens/16.4% clicks, respectively. Average session duration was 6:22 minutes.

“Yeah, I do feel that the emails gave a, um, a little, ‘Okay, you’ve had the information, you’ve had two days to integrate, you know, the next things and yep, this is your little booster or reminder,’ sort of thing, yeah that was good,”

Participant 10.

**Fig 3:**
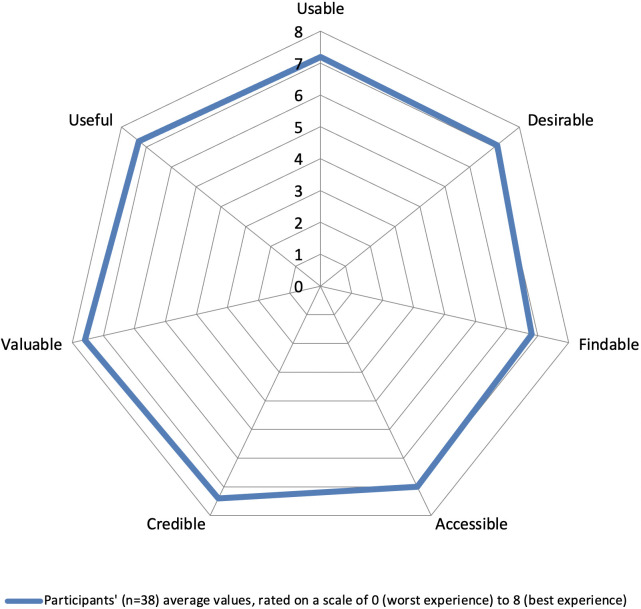
The User Experience Radar [26] with average values for the Honeycomb Model.

The self-administered questionnaires had a 95% response rate. The UES-SF domain scores were 130.6 for focused attention, 152.3 for aesthetic appeal, 154 for perceived usability and 169 for reward. The results for the Honeycomb Model showed positive overall outcomes across the seven domains, though the highest domain was valuable (Fig 3). Participants noted they valued their improved level of understanding carbohydrates and confidence in their ability to self-manage their T2D more effectively.

“Oh, the value, uh, well, how my diabetes is affecting me, um, and how I can manage it. That’s probably the biggest thing I’ve got out of it. Like before the course, I, you know, I, honestly, um, as far as management goes, there was none,”

Participant 1.

### Constructs of engagement

According to qualitative data, participants were initially motivated to engage with the intervention via affective responses. Participants expressed their motivation was prompted by the thought of avoiding medication and health risks associated with T2D. In addition, acceptance into the program increased their motivation, as they wanted to take advantage of the opportunity.

“I thought well I’ve been selected and, and I really appreciated that opportunity so I didn’t wanna stuff it up,”

Participant 13.

Cognitive processes then took place. Participants found it helpful to spend time familiarizing themselves with the program structure and content initially. Participants indicated the structured mode of delivery removed cognitive load and facilitated ease of use, even for those who declared they were technically challenged.

“I’m not super tech savvy and um, I’m learning this whole new way of doing things. But um, I, I was pretty pleased with myself. I actually learned stuff. I sort of didn’t grow up with all the technology, but um, yeah, I, I did it, so if I can do it, anyone can,”

Participant 5.

As participants cognitively consumed the intervention guidelines, behavioral responses followed. At their own pace and ability, participants implemented the program guidelines. The behavior changes noted were increasing vegetable intake, reading food labels, planning, preparation, problem solving, identifying food substitutes, and reducing junk food intake.

“I’m still monitoring what I’m doing on a regular basis, and planning meals in advance. Whereas before, well, we’re out of, out of food tonight, go down and get a hamburger or something like that. That doesn’t happen anymore, which is good. I’ve already got my breakfast ready for tomorrow morning when I go to work as well and, um, it’s the planning of it, you know, that I would never have done before. I would just come home and just slap two slices of bread and tomato sauce and devon (processed meat) or something on, or something like that. Now I plan what I’m gonna do,”

Participant 11.

As participants implemented the guidelines and program action steps, affective, cognitive and behavioral responses were combined. Participants were motivated to continue by feelings of success throughout the program and observable health outcomes. In particular, they noted that having the ability to access information when they needed it was useful and provided reinforcement.

“There was no pressure. It was just there when I needed it. And you know, I’d go back and look at things and, and re-listen, and re-read things. And you go, oh yeah, no, that is right. I, I did read that,”

Participant 5.

In addition, they noted repetition as important, as cognitively processing information and behaviorally implementing changes can take time.

“Following the, the weekly guidelines, uh, which is what I tried to do, as I said, it’s, it’s… I’m still processing. It’s gonna take me another three months to actually process all this stuff to a point where I’m getting to the place where I don’t really have to think about it,”

Participant 9.

Challenges noted by two participants included food cost, accommodating different household dietary needs, and a larger amount of weight loss than desired. One participant mentioned feeling too restricted with foods at times, one noted difficulty with motivation at different stages during the program. Yet even where challenges arose, participants indicated they were able to work through them and continue with the program to the best of their ability.

“When you’ve got to that eight-week point, and you said, "We’re halfway there," I thought, "Oh, I don’t think I can do this.” I mean we went through a (Covid-19) lockdown here as well during that time. So it was, it was like I felt like my life was being restricted…yeah, moving forward from here, I may well be okay to manage it. I mean I know I can look at something now and say, "No, there’s too many carbs in that. I’m not gonna eat it,”

Participant 12.

Participants frequently noted that engagement was facilitated through the weekly structured program delivery, described as “reassuring,” “looked forward to,” “each week, learning a little bit more,” “gave you time to think,” and it helped them to stay on track.

“If you didn’t have a weekly touch point, you, you could easily, yeah, drift away. But it was just that constant reminder. And after, probably after four or five weeks we were well, you know, on board and, and, um, just looking for the next week’s advice and we’d go from there,”

Participant 8.

## Discussion

This study was the first to use mixed methods to explore engagement with a web-based dietary program for people with T2D, which was developed via a user-centered approach. We believe the value of implementing a user-centered development approach carried through to the engagement outcomes presented above, with the results indicating positive engagement overall. During our development work [15], we were able to understand aspects of the user experience that were important to our target users. Consequently, many of the factors that appeared to facilitate positive engagement outcomes were related to areas where attention was focused during our user-centered development—weekly structured program delivery; relevant content; accessibility; and positively-framed easy to understand communication. For the first time, this study provided insight into the ways affective, cognitive and behavioral constructs may influence engagement. In addition, we were able to determine factors that contributed to effective engagement in this study. These insights will be further illuminated below.

Throughout consultation with participants, the structured delivery of the program was noted as a primary facilitator of engagement. Structured programs have previously been shown to improve T2D self-management [1, 35]. In face-to-face settings, 60% of randomized controlled trials delivering structured diabetes education programs have successfully improved behavioral and/or clinical outcomes [35]. In the context of a web-based dietary program, structure appears to facilitate engagement through reduced complexity and cognitive load, along with providing familiarity that fosters ease of use. Furthermore, in this study participants highlighted one of the major benefits of web-based education delivery—the need for ongoing reinforcement to support behavior change, an important feature of self-management support noted by the American Diabetes Association [1].

There have only been five previous web-based T2D dietary interventions evaluated using a randomized controlled trial [36]. Majority collected frequency metrics (login rates), while only two collected duration metrics. Duration of engagement was comparable, ranging from 6–12 minutes in previous studies and 6:22 minutes in the current study. Similar to these studies [36], frequency of engagement was highest within the first two weeks. This higher engagement seen early across interventions would be expected, as participants in the current study indicated that it took time for them to cognitively consume content initially. Previous studies however, saw waning frequency of engagement, with less than 47% of participants logging in at all from weeks 7–16 [37, 38]. In contrast, results from the current study showed sustained frequency of engagement in 80% of participants for the entire 16-week intervention.

Although reminders have been noted as an engagement prompt in web-based health interventions [39, 40], few previous online dietary interventions have used reminders as an engagement strategy [5]. In this study, the email open and click through rates, along with feedback from participants, provided good indication that the email notifications did facilitate improved frequency and intensity of engagement. Similar to previous online dietary interventions, the dropout attrition rate was lower than the nonusage attrition rate [5]. Dropout attrition (7.5%) and nonusage attrition (12.5%) were at the lower end compared to previous online dietary interventions of 3- to 6-month duration ranging from 3–26% and 11–29%, respectively [5].

Self-administered questionnaires are an effective tool for capturing post-intervention data, achieving a high response rate of 95% in this study, comparable to previous web-based T2D dietary interventions who achieved an 89% [41] and 92% [37] response rate. The UES-SF reward domain had the highest score. The reward domain is an indicator of intensity of engagement, as it relates to participants involvement and ability to apply an intervention in a real-world setting [19]. A previous online health-related study [42] found the UES-SF reward domain was the only domain associated with sustained engagement. This suggests that both frequency and intensity of engagement are necessary for effective engagement. The Honeycomb Model valuable domain had the highest score, which relates to the reward domain. Moreover, the UES-SF and Honeycomb Model displayed an overlap in positive outcomes for the usability domains. The outcomes of the questionnaires concur with participant feedback, which indicated that experience of value and reward and ease of use enhanced engagement in the behavior change process.

As proposed by previous authors, our exploration of the constructs of engagement indicated engagement shifts dynamically between engagement with the intervention and engagement in the behavior change process [2, 7]. One notable illumination however, was that the way participants felt, particularly in terms of positive affective states prior to and during the intervention, increased cognitive and behavioral engagement. In the context of this intervention, positive affective states were experienced by participants initially through acceptance into the program/study and perceived benefits of participation. Later, positive affective states prompted higher engagement via confidence in their ability to apply the guidelines and observable health outcomes. These insights are important, as they definitively highlight that engagement is more than just usage and more than just doing, as has been suggested by authors previously [10, 40]. Interestingly, qualitative data showed negative affective states such as waning motivation or dealing with challenges can also play a role in influencing behavior, something that has piqued the curiosity of previous authors [10]. There was indication this may reduce engagement, temporarily. However, there was also indication that participants can work through challenges and maintain enough engagement to obtain knowledge or skills that can still influence behavior. In this regard, the structured nature of the program was again a facilitator, as the frequent touch points and reminders supported participants to stay on track, which aligns with existing literature regarding structured program delivery and reminders, as noted above.

In terms of the characteristics of the intervention that contributed to effective engagement, the findings suggest it was predominantly the structured program delivery and accompanying email reminders, both of which provided ongoing reinforcement to participants. Similarly, a previous systematic review of web-based dietary interventions in T2D [36] found that structured program delivery with an intervention focused solely on dietary self-management, maintained engagement with low attrition rates. In contrast, interventions that focused on comprehensive self-management, were unstructured and overly complex, had low engagement and high attrition rates [36]. As indicated by the results of this study, structure and repetition reduces complexity and facilitates information uptake; while prompts improve frequency and intensity of engagement. There is currently a paucity of empirical evidence on the effectiveness of web-based dietary interventions in people with T2D, with very limited evidence of engagement outcomes [36]. The findings of this study in conjunction with the limited available evidence, suggest there needs to be greater emphasis on dietary self-management within web-based interventions to improve engagement in people with T2D. This observation is supported by numerous qualitative studies in people with T2D who have highlighted that food and nutrition support was a key area where extra assistance is required [43–46]. Furthermore, it is well understood that diet is the most challenging component of T2D self-management [1]. It is important to highlight, that users of an intervention are the direct line to improving the user experience to foster better engagement outcomes [47, 48]. Thus, employing a user-centered approach to intervention development [15] is recommended to improve engagement outcomes, as demonstrated by this study and previous web-based dietary interventions in T2D [41]. To further improve participant engagement and experience, findings of this study suggest exploring broader content (addressing costs/budgets and accommodating different household dietary needs), and the addition of a smartphone app may further benefit participants with T2D.

### Strengths and limitations

One of the major strengths of this study was the mixed method integration strategy to merge objective and subjective data for depth and breadth in understanding the complex topic of engagement. As a result of this approach, outcomes and insights have emerged that will be of value to current and future researchers using digital technologies to deliver lifestyle interventions for T2D or other chronic health conditions. There were some limitations in our methods, which is to be expected in this developing field of exploring engagement. Currently there are few validated engagement scales with widespread use [2]. Thus, it was challenging to make a comparison of the outcomes of the UES-SF and Honeycomb Model, as few digital health interventions have previously used these tools. However, the benefit of their use has been demonstrated in this study and can be used to explore/compare engagement in future interventions. The self-administered questionnaires and majority of usage data were collected anonymously as aggregate data, whereas it may have been useful to collect this data at an individual level to allow deeper exploration of the domains in comparison to frequency of use data, and an assessment of differences in terms of age-gender stratification. An assessment of data saturation in this study was based on pragmatism [13], information power [25] and interpretive judgement [18]. However, a more robust method for validating sample size during data collection has emerged [49] and would be applicable to future studies. While not necessarily a strength or limitation, it is important to note that this study was conducted during the height of the Covid-19 pandemic, which did impact the experiences of participants involved in the study and the qualitative data collected. Importantly, future web-based and digital studies in T2D need to measure and assess engagement, as there is currently very limited evidence available. Given this study has explored new territory, future research could look to replicate these methods and compare and contrast the outcomes to build further knowledge in this developing field of engagement.

## Conclusion

This study yielded novel findings by integrating qualitative and quantitative data to explore engagement with a web-based dietary program involving people with T2D. The results showed positive overall engagement, with frequency and intensity of engagement sustained throughout the program. The constructs of engagement were intricately linked, though engagement was enhanced through positive affective states, influencing participant’s cognitive and behavioral engagement and their experience of value and reward. In addition, the structured weekly mode of delivery was a key facilitator of engagement. Importantly, user-centered development conducted prior to intervention delivery provided the foundations for achieving positive engagement outcomes. The engagement outcomes that emerged as a result of this mixed methods exploration have advanced the field by providing a more comprehensive understanding of the complexities of engagement.

## Supporting information

S1 Checklist COREQ (COnsolidated criteria for REporting Qualitative research) checklist. (PDF)

S1 Appendix Self-administered questionnaires. (PDF)

S2 Appendix Interview guide. (PDF)

S3 Appendix A priori coding tree. (PDF)

## Abbreviations

T2D: type 2 diabetes
UES-SF: User Engagement Scale-short form
