# Supplementary material for: Exploring engagement with a web-based dietary intervention for adults with type 2 diabetes: A mixed methods evaluation of the T2Diet study
Source: PLoS One. 2022 Dec 30;17(12):e0279466. doi: 10.1371/journal.pone.0279466 (PMC9803196; doi:10.1371/journal.pone.0279466)
Supplement: S1 Appendix — (PDF) [file pone.0279466.s002.pdf]

S1 Appendix: Self-administered questionnaires

User Engagement Scale - short-form (UES-SF)

Please read the statements below and rate your experience on a scale of 1 to 5.

1=strongly disagree | 5=strongly agree

1. I lost myself in this experience.
2. The time I spent using the T2Diet Program just slipped away.
3. I was absorbed in this experience.
4. I felt frustrated while using the T2Diet Program.
5. I found the T2Diet Program confusing to use.
6. Using the T2Diet Program was taxing.
7. The T2Diet Program was attractive.
8. The T2Diet Program was aesthetically appealing.
9. The T2Diet Program appealed to my senses.
10. Using the T2Diet Program was worthwhile.
11. My experience was rewarding.
12. I felt interested in this experience.

The Honeycomb Model

Please rate your experience on a scale of 1 to 8.

0=worst experience; 8=best experience

1. How useful did you find the T2Diet Program in helping you learn about a reduced carbohydrate diet for the management of type 2 diabetes?
2. How easy did you find the website to use?
3. How much did you enjoy participating in the T2Diet Program?
4. How easy did you find the website to navigate when you were looking for information or resources?
5. Did you find the resources provided during the program were easy to understand?
6. How credible and trustworthy did you find the information and resources provided to you?

7. How valuable did you find the information and resources provided to you?

Exploring engagement with a web-based dietary intervention for adults with type 2 diabetes: a mixed methods evaluation of the T2Diet study

Jedha Dening[1], Karly Zacharia[2], Kylie Ball[1#], Elena S George[1#], Sheikh Mohammed Shariful Islam[1#]

[1] Institute for Physical Activity and Nutrition, School of Exercise and Nutrition Sciences, Deakin University, Locked Bag 20000, Geelong, Victoria, 3220, Australia

[2] Faculty of Health & Medicine, School of Health Sciences, University of Newcastle, Callaghan, NSW 2305, Australia

[#]equal contribution
