# Supplementary material for: Exploring engagement with a web-based dietary intervention for adults with type 2 diabetes: A mixed methods evaluation of the T2Diet study
Source: PLoS One. 2022 Dec 30;17(12):e0279466. doi: 10.1371/journal.pone.0279466 (PMC9803196; doi:10.1371/journal.pone.0279466)
Supplement: S2 Appendix — (PDF) [file pone.0279466.s003.pdf]

S2 Appendix: Interview Guide

What did you think of the program?

Thinking back to week one when you came into the program, how did you use the program guidelines, tools and resources?

What particular things about the program did you find most useful?

How useful was the program in supporting you in terms of various situations such as family or social situations or times you were away from home?

If there was something that you could change or add to the program, what would it be?

How motivated did you feel to use the program these past 16 weeks?

How did the program make you feel in terms of managing your type 2 diabetes?

Did you find the program and resources credible and trustworthy? What elements of the program fostered that trust/distrust for you?

What was your experience finding your way around the program?

Can you describe what you thought about the way everything was communicated to you?

What are your thoughts about the overall presentation of the program?

What would you consider the most valuable takeaways from being a part of the T2Diet program?

Do you recall anything else about your experience that you would like to share?

Exploring engagement with a web-based dietary intervention for adults with type 2 diabetes: a mixed methods evaluation of the T2Diet study

Jedha Dening[1], Karly Zacharia[2], Kylie Ball[1#], Elena S George[1#], Sheikh Mohammed Shariful Islam[1#]

[1] Institute for Physical Activity and Nutrition, School of Exercise and Nutrition Sciences, Deakin University, Locked Bag 20000, Geelong, Victoria, 3220, Australia

[2] Faculty of Health & Medicine, School of Health Sciences, University of Newcastle, Callaghan, NSW 2305, Australia

[#]equal contribution
