# Supplementary material for: Exploring engagement with a web-based dietary intervention for adults with type 2 diabetes: A mixed methods evaluation of the T2Diet study
Source: PLoS One. 2022 Dec 30;17(12):e0279466. doi: 10.1371/journal.pone.0279466 (PMC9803196; doi:10.1371/journal.pone.0279466)
Supplement: S3 Appendix — (PDF) [file pone.0279466.s004.pdf]

**S3 Appendix: *A Priori* Coding Tree**

| | |
|---|---|
| **Behavioral** | Reactions or actions made in response to program stimuli, with an interest to learn more about offline engagement |
| **Affective** | Relating to feelings, attitudes and moods (positive or negative) |
| **Cognitive** | Process of acquiring knowledge and understanding, making decisions and producing responses |
| **Intervention features, content, mode of delivery** | Exploring intervention features: eg, what is it about 'X' that was so appealing? Impacted intentions, etc? |
| **Behavior changes** | Most common behavior changes/ patterns for participants |

**Exploring engagement with a web-based dietary intervention for adults with type 2 diabetes: a mixed methods evaluation of the T2Diet study**

Jedha Dening[1], Karly Zacharia[2], Kylie Ball[1#], Elena S George[1#], Sheikh Mohammed Shariful Islam[1#]

[1] Institute for Physical Activity and Nutrition, School of Exercise and Nutrition Sciences, Deakin University, Locked Bag 20000, Geelong, Victoria, 3220, Australia

[2] Faculty of Health & Medicine, School of Health Sciences, University of Newcastle, Callaghan, NSW 2305, Australia

[#]equal contribution
